# Optical imaging of gastric cancer with near-infrared heptamethine carbocyanine fluorescence dyes

**DOI:** 10.18632/oncotarget.10031

**Published:** 2016-06-14

**Authors:** Ningning Zhao, Caiqin Zhang, Yong Zhao, Bing Bai, Jiaze An, Hai Zhang, Jason Boyang Wu, Changhong Shi

**Affiliations:** ^1^ Laboratory Animal Center, the Fourth Military Medical University, Xi'an, Shaanxi 710032, China; ^2^ Uro-Oncology Research Program, Samuel Oschin Comprehensive Cancer Institute, Department of Medicine, Cedars-Sinai Medical Center, Los Angeles, CA 90048, USA

**Keywords:** near-infrared fluorescence, heptamethine carbocyanine dyes, gastric cancer, patient-derived tumor xenograft, HIF1α

## Abstract

Near-infrared fluorescence (NIRF) imaging agents are promising tools for noninvasive cancer imaging. Here, we explored the tumor-specific targeting ability of NIRF heptamethine carbocyanine MHI-148 dye in cultured gastric cancer cells, gastric cancer cell-derived and patient-derived tumor xenograft (PDX) models. We show that the NIRF dye specifically accumulated in tumor regions of both xenograft models, suggesting the potential utility of the dye for tumor-specific imaging and targeting in gastric cancer. We also demonstrated significant correlations between NIRF signal intensity and tumor volume in PDX models. Mechanistically, the higher cellular uptake of MHI-148 in gastric cancer cells than in normal cells was stimulated by hypoxia and activation of a group of organic anion-transporting polypeptide (OATP) genes. Importantly, this NIRF dye was not retained in inflammatory stomach tissues induced by gastric ulcer in mice. In addition, fresh clinical gastric tumor specimens, when perfused with NIR dye, exhibited increased uptake of NIR dye *in situ*. Together, these results show the possibility of using NIRF dyes as novel candidate agents for clinical imaging and detection of gastric cancer.

## INTRODUCTION

Near-infrared fluorescence (NIRF) dyes are a class of polycyanine heterocyclic compounds that are formed with large π-conjugated systems composed of heterocyclic rings or aromatic rings at both ends or in the middle and intramolecular polymethine chains [1]. Because of low autofluorescence, tissue absorbance and scattering at NIR wavelengths (700-1,000 nm), these dyes are excellent imaging probes as demonstrated in both animals and humans [2, 3]. With good tissue permeability and the deep penetration of absorbed NIRF in biological tissues, these dyes have shown great potential for noninvasive imaging and tumor targeting [4–6]. We previously identified and synthesized a unique group of NIRF heptamethine carbocyanine dyes as dual imaging and targeting agents, including MHI-148 and IR-783 [7, 8]. These dyes could be specifically used for direct recognition of human genitourinary tumor cells, and showed preferential uptake in tumor cells but not normal cells in a variety of preclinical and clinical model systems [9, 10]. These agents can also undergo a number of chemical modifications, including conjugation with effective therapeutic drugs to bind cancer cells at different stages [4, 11, 12]. The dye uptake is stimulated in part by tumor hypoxia and activated hypoxia-inducible factor 1α (HIF1α)/organic anion-transporting polypeptide (OATP) signaling [7, 13]. However, whether these dyes possess the potential to bind gastric cancer and associated malignant events remains to be explored.

The patient-derived tumor xenograft (PDX) model is constructed by transplanting fresh patient tumor tissues into immunodeficient mice, which well preserves the basic characteristics of the primary tumor as well as the microenvironment [6, 14, 15]. The PDX models also largely maintain the heterogeneity and complexity of human tumors by recapitulating the biological features of gene expression and mutational status at the molecular level, providing an excellent *in vivo* model for cancer research [15]. However, the establishment of PDX models is time-consuming, with an observation period of more than two months prior to confirmed xenograft growth in most cases, and also lacks reliable and efficient imaging methods for xenograft recognition [16]. Therefore, there is a growing need for developing imaging probes with high specificity and sensitivity to visualize tumor xenografts in PDX models to advance current cancer research.

In this study, we utilized a number of *in vitro* and *in vivo* gastric tumor models, including tumor xenografts originating from cultured cancer cells and PDX models, to investigate the binding potential of a group of NIRF agents, represented by MHI-148 dye and its dye-drug derivative, in gastric cancer. We also explored the accompanying molecular mechanisms.

## RESULTS

### Preferential accumulation of MHI-148 in gastric cancer cells

**Figure 1:**
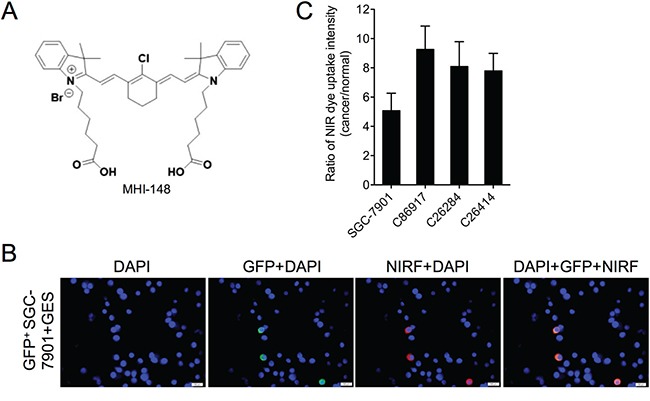
Uptake of MHI-148 dye by human gastric cancer cells **A.** Chemical structure of MHI-148. **B.** NIRF imaging of gastric normal-cancer cell co-cultures. MHI-148 dye (5 μM, 10 min) was incubated with GFP-tagged human gastric cancer SGC-7901 cells co-cultured with normal human gastric epithelial GES cells. Nuclei from both SGC-7901 and GES cells were stained by DAPI. Scale bars represent 50 μm. **C.** Ratio of NIRF dye uptake intensity in different human gastric cancer cell lines as compared to human normal gastric epithelial GES cells. Data are presented as the mean ± SD (n=5).

To determine whether the NIRF dye specifically targets gastric cancer cells but not normal gastric cells, we established an *in vitro* co-culture model in which human gastric cancer SGC-7901 cells dually labeled with both green fluorescence protein (GFP) and luciferase (luc) were cultured with normal human gastric epithelial GES cells. Lentiviral infection-mediated GFP labeling of SGC-7901 cells followed by puromycin selection ensured a 100% integrated rate of GFP in stable SGC-7901 cells, which was demonstrated by fluorescence microscopy (data not shown). To examine the dye uptake, the co-culture was incubated with MHI-148 (chemical structure shown in Figure 1A) and subjected to fluorescence microscopy. The NIRF signal was exclusively observed in GFP-positive SGC-7901 cells but not the other GFP-negative GES cells (Figure 1B), suggesting the preferential uptake and retention of MHI-148 in gastric cancer cells but not normal cells. We also examined the dye uptake in this co-culture model by replacing SGC-7901 cells with three cultured cancer cell lines derived from three different PDX models, including C86917, C26284 and C26414, and observed dye uptake in a cancer cell-specific manner (data not shown). Quantitative analysis further revealed an up to 9-fold increase of dye uptake in different gastric cancer cells compared to normal gastric cells (Figure 1C), indicating the specific uptake of MHI-148 dye by gastric cancer cells.

### Correlation of MHI-148 dye uptake with gastric tumor xenograft growth

**Figure 2:**
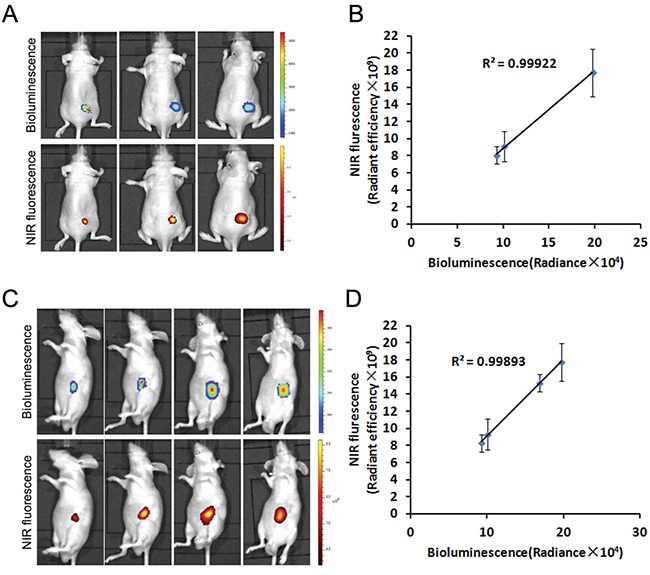
Preferential uptake and retention of MHI-148 dye in human gastric cancer subcutaneous and orthotopic xenograft models **A.** Dual BLI/NIRF imaging of SGC −7901 cell-derived tumor xenografts in subcutaneous models. Different numbers of luc-tagged SGC-7901 cells were injected into nude mice to establish subcutaneous tumor xenografts. Two weeks later, tumor-emitting signals by both BLI and NIRF imaging were captured. Representative images are shown. **B.** The correlation of BLI/NIRF signal intensity in A was determined (n=5 for each group). The mean was used to plot the correlation curves. **C.** Dual BLI/NIRF imaging of SGC-7901 cell-derived tumor xenografts in orthotopic models. Luc-tagged SGC-7901 cells were injected in the stomach serosa of nude mice to establish orthotopic xenografts. BLI and NIRF signals in mice subjected to both imaging modalities were detected on Day 14, 19, 24 and 29. Representative images are shown. **D.** The correlation of BLI/NIR signal intensity in C was determined (n=5 for each group). The mean was used to plot the correlation curves.

To determine whether the preferential uptake of MHI-148 by gastric cancer cells *in vitro* could be recapitulated *in vivo*, we implanted different numbers (1 × 10^6^, 3 × 10^6^ and 9 × 10^6^) of luc-tagged SGC-7901 cells into nude mice to establish subcutaneous tumor xenograft models (Figure 2A). We also took a similar approach to establish orthotopic xenograft models by injecting luc-tagged SGC-7901 cells directly in the stomach serosa of nude mice (Figure 2C). Two weeks later, MHI-148 was shown to be specifically accumulated in tumor xenografts in both models as demonstrated by whole-body NIRF imaging (Figure 2A and 2C). This was further supported by co-registered bioluminescence (BLI) and NIRF signals in tumors (Figure 2A and 2C), with a significant positive correlation of signal intensity between the two imaging modalities in both models (Figure 2B and 2D). These results suggest the ability of MHI-148 to specifically bind gastric tumors in xenograft models and more importantly to quantitatively estimate tumor growth profiles *in vivo*.

### Uptake of NIRF dye and dye-drug conjugate in gastric cancer PDX models

**Figure 3:**
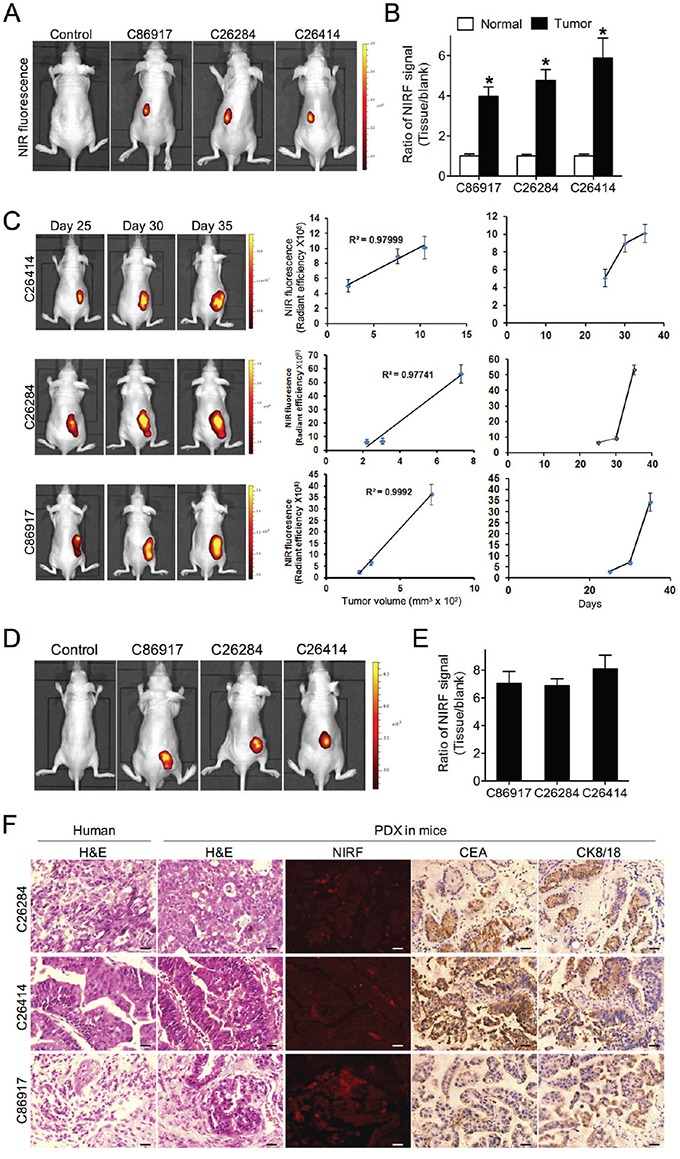
Uptake of NIRF dye and dye-drug conjugate in gastric cancer PDX models **A.** NIRF imaging of PDX models established by implanting 3 different human fresh gastric cancer specimens to one-side subrenal capsules of nude mice. Mice without implantation were used as control. Representative images are shown. **B.** Quantification of MHI-148 dye uptake in both normal kidney and subrenal tumor xenografts from PDX models. Data are presented as the ratio of dye uptake intensity as normalized to blank region (mean ± SD, n=5). *p<0.05. **C.** The correlation of tumor-emitting NIRF signal intensity with tumor size from 3 PDX models as measured at 3 different time points (mean ± SD, n=5). Representative images are shown. The mean was used to plot the correlation curves. **D.** NIRF imaging of IR-783-gemcitabine (NIRG) uptake in 3 different human gastric cancer PDX models. Mice without implantation were used as control. Representative images are shown. **E.** Quantification of NIRG dye-drug uptake in D. Data are presented as the ratio of dye uptake intensity as normalized to blank region (mean ± SD, n=5). **F.** H&E, NIRF and IHC analyses of tumor tissues derived from both PDX mouse models and original patient samples. Original magnification, ×400; scale bars represent 20 μm.

To test the dye uptake in PDX models, we implanted 5 gastric cancer patient samples into the subrenal capsules of nude mice to establish PDX models, and were able to detect xenograft growth with 3 samples, including C26284, C26414 and C86917. The histology subtypes of 3 gastric tumors were poorly-differentiated adenocarcinoma, well-differentiated adenocarcinoma and mucous adenocarcinoma respectively. At 6 weeks, significant NIRF signals with up to a 6-fold increase were demonstrated in all 3 PDX-derived tumor xenografts compared to the marginal light detected in normal kidneys of both PDX-bearing mice and control mice (Figure 3A and 3B). We were further able to capture the NIRF signal in tumor sections by fluorescence microscopy (Figure 3F and Supplemental Figure 1). Moreover, tumor-emitting NIRF signals increased along with xenograft growth, with a significant positive correlation of signal intensity with tumor volume as measured by caliper in all 3 PDX models (Figure 3C). Mice bearing a subcutaneous PDX-derived gastric tumor were injected with MHI-148 intraperitoneally at a dose of 50 nmol/mouse. 24 hr later, the dye uptake in tumor tissue was significantly higher than other vital organs (Supplemental Figure 2). In addition, we examined the targeting potential of IR-783-gemcitabine (NIRG), a dye-drug conjugate chemically linking IR-783 dye, a MHI-148 analog, with the chemotherapeutic drug gemcitabine [12, 17], in PDX models. As shown by whole-body NIRF imaging, NIRG exhibited specific accumulation in subcutaneous xenografts compared to normal tissue from both PDX-bearing mice and control mice (Figure 3D and 3E). These results suggest that these NIRF dyes could be used as drug carriers to deliver therapeutic agents specifically to tumors in gastric cancer PDX models. Finally, we confirmed an identical histology in PDX-derived tumor tissues compared to the original patient's tumor samples by H&E staining, which was accompanied by the strong expression of CEA and CK8/18, two well-expressed markers in gastric cancer, in PDX samples (Figure 3F). These results support the notion that the PDX-derived tumors well maintain the morphology and the principal molecular characteristics of primary gastric tumors [6, 14]. In conclusion, we demonstrated that these NIRF dyes and derivative NIRF dye-drug conjugates could be used to visualize tumor xenografts in gastric cancer PDX models with sufficient specificity and sensitivity.

### MHI-148 dye uptake in gastric cancer cells is cooperatively stimulated by tumor hypoxia and OATPs

We previously reported that the cancer-specific uptake of NIRF dye could be partially mediated by tumor hypoxia and the activation of a group of cell membrane-bound OATPs in a number of genitourinary cancer models [7, 13]. Hypoxia is frequently observed in a spectrum of solid tumors, including gastric tumor, and affects gene expression and cell behavior mainly through HIF1α, a master regulator of hypoxia signaling [18]. On the other hand, OATPs represent a superfamily of solute carrier transporters, including 11 known human OATPs classified into 6 families and subfamilies, which facilitate the transport of a number of substances, including organic acids, drugs and hormones, into cells in a highly substrate- and pathophysiologic-dependent manner [19]. Recent evidence has further indicated the association of dysregulated expression of OATPs with cancer development, including gastric cancer [19–21].

**Figure 4:**
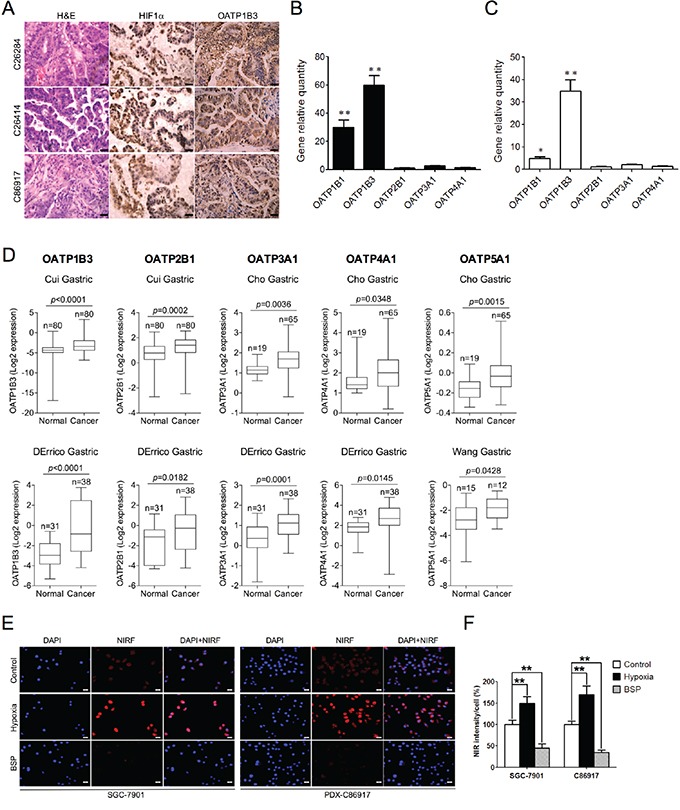
Mechanisms of NIRF dye uptake by gastric tumor cells and xenografts **A.** H&E and IHC analyses of HIF1α and OATP1B3 protein expression in gastric tumor tissues derived from 3 PDX models. Original magnification, ×400; scale bars represent 20 μm. **B.** qPCR analysis of select *OATPs* in PDX-derived tumor tissues. Data are presented as the fold change (mean ± SD, n=10) of gene expression in tumor tissues as compared to normal gastric tissues. **p<0.01. **C.** qPCR analysis of select *OATPs* in gastric cancer cells. Data are presented as the fold change (mean ± SD) of gene expression as compared to normal GES cells. **p<0.01. **D.** Oncomine analysis of select *OATPs* transcript levels in different gastric cancer data sets (normal vs. cancer). **E.** NIRF imaging of *in vitro* uptake of MHI-148 (5 μM, 10 min) by both SGC-7901 and PDX-derived C86917 cells under pre-treatment with either hypoxia (1% O_2_, 1 hr) or bromosulfophthalein (BSP) (250 μM, 1 hr). Representative images are shown. Scale bars represent 50 μm. **F.** Quantification of NIRF dye uptake in E. Intensity in control cells under normoxia and without treatment was set as 100% (mean ± SEM, n=5). **p<0.01.

Given these common features shared by gastric cancer and other types of cancers, such as tumor hypoxia and aberrant OATP expression, we extended our previous findings to examine whether gastric cancer cells may use similar mechanisms to take up and retain NIRF dyes. To test this idea, we analyzed the expression levels of HIF1α and OATP1B3, a representative member of OATP family, in PDX-derived tumor tissues, and found high expression of both proteins, with HIF1α and OATP1B3 expressed in the nucleus and cytoplasm of tumor cells respectively in all 3 PDX samples (Figure 4A). Next, we surveyed the expression of various OATPs in 10 clinical gastric cancer specimens together with paired normal gastric tissues by qPCR. As shown in Figure 4B, 5 *OATPs* showed higher mRNA expression, with the most obvious increases seen in *OATP1B1* and *OATP1B3* in tumor tissues compared to relative normal tissues. Similar observations were also made in cultured gastric cancer cells, with the highest fold induced for the expression of *OATP1B3*, compared to normal gastric GES cells (Figure 4C). We further analyzed a number of human gastric cancer data sets deposited at the Oncomine website, a cancer microarray database and data-mining platform [22], and identified significantly higher expression of select *OATPs*, including *OATP1B3, OATP2B1, OATP3A1, OATP4A1* and *OATP5A1*, in gastric cancer patient samples compared to normal tissue samples (Figure 4D). To study whether tumor hypoxia and highly-expressed *OATPs* directly increase dye uptake in gastric cancer cells, we treated human gastric cancer SGC-7901 and gastric cancer PDX-derived C86917 cells with either a hypoxic stimulus (1% O_2_) or bromosulfophthalein (BSP), a competitive inhibitor of OATPs, prior to dye exposure. Our results showed that hypoxic stimuli led to significant increases of dye uptake, whereas cells pre-treated with BSP showed reduced dye uptake in both cell lines (Figure 4E and 4F). These results in sum suggest the mediating role of both tumor hypoxia and activation of OATPs in dye uptake by gastric cancer cells.

### Differential uptake of NIRF dye in gastric cancer and gastritic tissues

**Figure 5:**
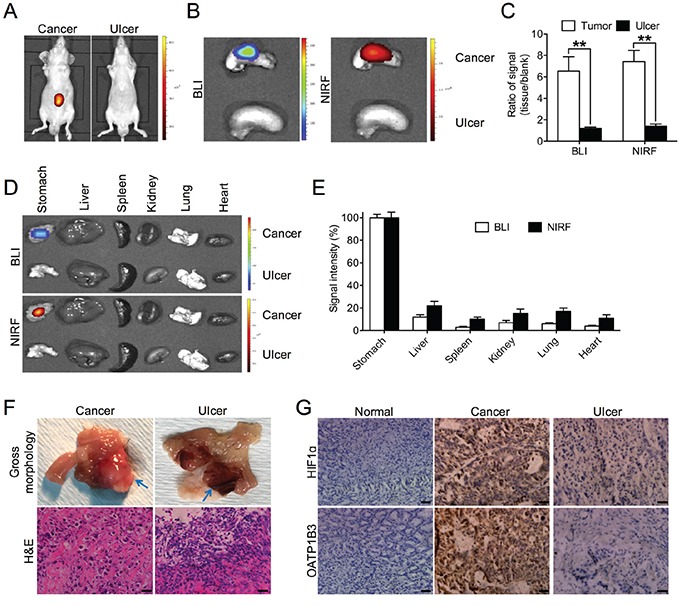
Preferential uptake of NIRF dye in gastric cancer relative to gastritic tissues **A.** *In vivo* NIRF imaging of mice bearing either orthotopic luc-tagged gastric tumor xenografts (left) or gastric ulcer (right). **B.** *Ex vivo* dual BLI/NIRF imaging of mouse stomach in A. **C.** Quantification of B for both modalities. Data are presented as the ratio (mean ± SD, n=5) of BLI/NIRF signal intensity as compared to blank region. **p<0.01. **D.** *Ex vivo* dual BLI/NIRF imaging of select organs, including the stomach, liver, spleen, kidney, lung and heart, as dissected from mice in A. **E.** Quantification of both BLI and NIRF signal intensity from the tumor-bearing experimental group in D. Data are presented as the percentage (mean ± SD, n=5) of signal intensity as normalized to stomach. Signal intensity in stomach is set as 100%. **F.** Gross morphology and H&E staining of gastric cancer and ulcer tissues in A. Representative images are shown. Blue arrows indicate tumor (left panels) and ulcer (right panels) area respectively. Original magnification, ×400; scale bars represent 20 μm. **G.** IHC analysis of HIF1α and OATP1B3 expression in gastric normal, cancer and ulcer tissues. Representative images are shown. Original magnification, ×400; scale bars represent 20 μm.

A critical issue in clinical diagnosis of gastric cancer using imaging approaches is to clearly differentiate gastric cancer from gastritis, a pathologic phenomenon commonly associated with the development of gastric cancer [23, 24]. To study how gastritis responds to the NIRF dye, we established a gastritic model by inducing gastric ulcer to mimic stomach inflammation in mice, in parallel with orthotopic tumor xenograft models using luc-tagged SGC-7901 cells. By exposing the mice from both gastric tumor and ulcer groups to the NIRF dye, we demonstrated strong NIRF signals clearly captured from gastric tumors in contrast to undetectable fluorescence in stomach ulcer by whole-body NIRF imaging (Figure 5A). Similar observations were also made in the excised stomach organ by both BLI and NIRF imaging modalities through an *ex vivo* approach (Figure 5B). Quantitative analysis indicated a 6-fold drop of signal intensity in ulcerous stomach relative to cancerous stomach for both BLI and NIRF modalities (Figure 5C). Moreover, the accumulation of MHI-148 dye was found to be specific to tumor-bearing stomach but not ulcerous stomach or other normal major organs dissected from mice, including the liver, spleen, kidney, lung and heart, by both BLI and NIRF imaging (Figure 5D), with a 5- to 10-fold increase of BLI/NIRF signal intensity in cancerous stomach compared to the others in the gastric tumor experimental group (Figure 5E). In addition, we confirmed the different types of gross morphology and histology present in both tumor and ulcer tissues by H&E staining (Figure 5F). To explore the contrasting uptake responses to the NIRF dye as exhibited by gastric tumor and ulcer tissues, we analyzed HIF1α and OATP1B3 expression, which are considered to increase dye uptake in gastric cancer cells, in different tissue samples. Compared to strong expression of both proteins in tumor tissues, ulcerous tissues demonstrated only marginal expression of both HIF1α and OATP1B3. As expected, negative expression was found in normal gastric tissues (Figure 5G). Together, these results by parallel comparisons indicate the potential unique use of NIRF dye for the detection of gastric tumors based on its minimum interference with gastritis and other inflammatory events in the stomach.

### NIRF imaging of clinical gastric cancer samples *in situ*

**Figure 6:**
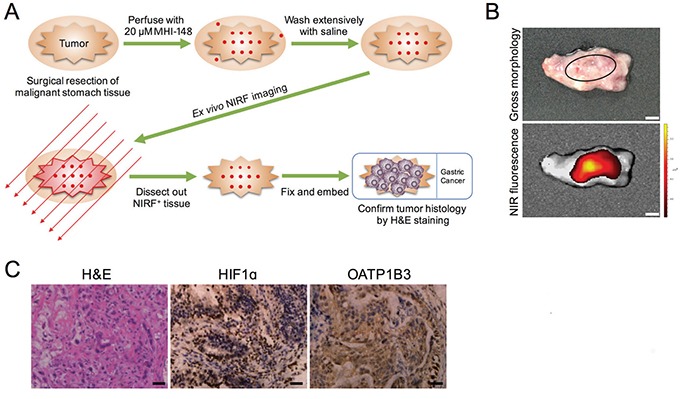
NIRF imaging of clinical gastric cancer samples **A.** Schematic outlining the experiment procedures for NIRF imaging of freshly resected clinical gastric tumor samples. **B.** Gross morphology and NIRF imaging of tumor tissues surgically resected from a representative case of three gastric cancer patients. Representative images are shown. Original magnification: x4; scale bars represent 4 mm. **C.** H&E and IHC analyses of HIF1α and OATP1B3 expression in tumor samples in B. Representative images are shown. Original magnification: x400; scale bars represent 20 μm.

To study whether the preferential uptake of NIRF dye by gastric cancer as revealed in a series of preclinical models could be directly recapitulated in clinical samples, we applied MHI-148 dye to detect gastric tumors surgically removed from three patients by perfusion (Figure 6A). As demonstrated by *ex vivo* NIRF imaging, the tumor areas in excised gastric tissues from all three cases were specifically detected by MHI-148 dye in sharp contrast to the unobservable signal in the nearby associated normal counterpart tissues (Figure 6B). We further dissected out NIRF-positive tissues and confirmed tumor histology by H&E staining in these samples (Figure 6C). Importantly, we showed intense widespread nuclear HIF1α and cytoplasmic OATP1B3 expression in NIRF-positive tumor samples (Figure 6C). These clinical observations reinforce the cancer-specific targeting ability of NIRF dye as well as the activate roles of HIF1α/OATPs in mediating dye uptake in gastric cancer.

## DISCUSSION

In this study, we demonstrated for the first time the specific uptake of a group of NIRF heptamethine carbocyanine dyes by gastric cancer cells and xenograft models. These dyes possess dual imaging and targeting properties without the need for chemical conjugation [4, 8]. We showed that the MHI-148 dye was taken up and accumulated specifically in human gastric cancer cells and xenograft tumor tissues as examined by NIRF microscopy and an optical imaging system, respectively (Figures 1 and 2). The NIRF signal was observed exclusively in GFP-positive human gastric cancer cells but not in GFP-negative normal human gastric epithelial cells. Similar results were also found in cultured cancer cells derived from three PDX samples, indicating the ability of MHI-148 to recognize clinically-mimicking gastric cancer cells (Figure 1). In addition, a significant linear correlation between BLI and NIRF signal intensity in tumor xenografts was obtained in both subcutaneous and orthotopic implantation models, which reinforces the preferential dye uptake by cancer cells relative to normal cells in a quantitative manner (Figure 2). These results in aggregate confirm the tumor-specific targeting ability of MHI-148 dye in gastric tumor cells and xenografts.

It is well known that tumor heterogeneity is common to nearly all types of cancers. Conventional tumor xenograft models are established through the transplantation of cancer cells into immunodeficient mice to produce cell line-derived xenograft (CDX) models. However, the cultured cancer cells lose most of their primary tumor tissue specificity and tumor homogeneity in order to adapt to a microenvironment *in vitro*. Thus, the CDX models are largely different from clinical patient samples, leading to difficulty applying most preclinical experimental results from CDX models to clinical practice [6, 25, 26]. Also, traditional tumor NIRF imaging agents require chemical conjugation of imaging probes with particular tumor-specific ligands, such as metabolic substrates, growth factors and antibodies [27–29]. Consequently, only a very few specific types of cancer cells in CDX models can be detected by traditional NIRF imaging approaches [30].

By maintaining perfect comparability with patients' tumors, the PDX model shows exciting prospects for screening and testing anticancer drug efficacy tailored for individualized treatment [31]. Subcutaneous implantation of patient tumor tissues into nude mice is a conventional method to construct a PDX model for easy observation, though the success rate can be as low as 25% and even worse when implanted tumors display a low degree of malignancy and an early tumor stage [32]. By contrast, the subrenal capsule is a good site for tumor implantation in PDX models, with a more than 70% success rate, since it has abundant blood supply as well as fine histocompatibility [33]. Nevertheless, the subrenal capsule is not easily observed after tumor formation. With the introduction of these heptamethine carbocyanine dyes, we were able to visualize gastric PDX tumors by NIRF imaging with stable capture even in subrenal xenografts in deep tissues (Figure 3). These imaging probes are a significant advance over previous early screening and evaluation PDX models *in vivo*, providing a much more effective NIRF imaging method. Our results demonstrated the detection of obvious fluorescence signals only 6 weeks after subrenal capsule tumor implantation (Figure 3), whereas routine subcutaneous implantation takes more than 2 months to determine a successful growth outcome. Among a total of five gastric cancer specimens, three were xenografted successfully through the subrenal capsule implantation and stably passaged to P3 generation in nude mice, a much higher success rate than the subcutaneous route, as evidenced by optical fluorescence imaging (Figure 3).

Through continual NIRF imaging processes, the intensity of the fluorescence signal emitted by tumor xenografts was well correlated with tumor volume, and the tumor growth curves of gastric cancer PDX models were also consistent with the biological features of tumor cell proliferation. Tumor xenograft tissues maintained the characteristics of the original tumors as examined by H&E staining and IHC analysis of select markers, such as CEA and CK8/18. In addition, we demonstrated that both MHI-148 and NIRG accumulated specifically in the tumor regions of gastric cancer PDX models (Figure 3). These observations show that these dyes and dye-drug conjugates hold promise for further exploration as imaging and targeting agents for the treatment of gastric cancer.

A precise diagnosis of gastric cancer versus gastritis is important in clinical practice, particularly given the symbiotic presence of these two pathologic entities in a significant portion of early-stage gastric cancer patients [23, 24]. In this study, we demonstrated that MHI-148 dye could well differentiate gastric cancer from gastritis, which is supported by significantly higher dye accumulation in gastric tumor regions compared to the minimal dye uptake by inflammatory stomach tissues induced by gastric ulcer in mice (Figure 5). We also demonstrated specific retention of NIRF dye in clinical gastric cancer resection specimens, without detecting any association of NIRF dye uptake with gastritic areas by histological analysis (Figure 6). These results further reinforce the potential of these heptamethine carbocyanine dyes as a promising and reliable tool for gastric cancer imaging in clinical scenarios. Crucial safety evaluations of this NIRF dyes are needed in mouse and dog before clinical use. Furthermore, this dye coupling with different imaging modalities such as near infrared ray electronic gastroscopy may be a promising application in the clinic for gastric cancer diagnostics.

The mechanisms underlying the uptake of NIRF dyes in cancer cells have been previously reported to be increased by tumor hypoxia and activation of the HIF1α/OATPs signaling axis [7, 13]. Altered OATP expressions and variants have been implicated in many different types of cancers by several groups [19–21]. In this study, we showed for the first time a comprehensive survey indicating that the expression of select *OATPs*, including *OATP1B3, OATP2B1, OATP3A1, OATP4A1* and *OATP5A1*, is correlated with human gastric cancer progression as examined in a number of human clinical data sets, which provides a rationale for the role of these transporters in increasing NIRF dye uptake in gastric cancer cells (Figure 4). Human gastric cancer cells derived from both cell culture and PDX models significantly increased dye uptake under hypoxic conditions but retained less dye after pre-treatment with BSP, a competitive OATP inhibitor (Figure 4). Moreover, both HIF1α and OATP1B3 were strongly expressed in all three PDX tumor samples and their original clinical gastric tumor specimens (Figures 4 and 6). These observations are in agreement with our previous findings in genitourinary and brain cancer models [12, 13]. In addition to these insights from the cooperative actions of hypoxia and OATPs, we speculated that alternative mechanisms might also participate in the regulation of NIRF dye uptake in gastric cancer, which is supported by the observation that BSP did not completely block dye uptake in cells (Figure 4). For example, NIRF dye Pz 247 was reported to associate with the lipophilic core of LDL and undergo cellular entry primarily through receptor-increased endocytosis to accumulate in lysosomes. Given that LDL-bound cholesterol also exists in highly proliferative gastric tumor cells [34], further investigations are warranted to better understand the mechanisms underlying the uptake of NIRF dyes in gastric cancer.

In this study, the NIRF MHI-148 dye was successfully applied for the optical imaging of gastric cancer cell xenograft and PDX models, and was quantitatively correlated with tumor growth profiles. This dye could also serve as a drug carrier to deliver a chemotherapy drug to the tumor center in PDX models for therapeutic purposes. The molecular mechanisms underlying the preferential uptake of NIRF dyes by gastric cancer cells primarily involve tumor hypoxia and activation of select *OATP* genes, such as *OATP1B3*. Importantly, we demonstrated increased uptake of MHI-148 *in situ* in perfused clinical gastric tumor samples, which did not interfere with gastritis commonly associated with gastric cancer. Together, these preclinical results support the idea of that further developing this group of NIRF dyes along with coupled fluorescence imaging methods would have clinical utility in cancer imaging and targeting and could be adopted in the near future.

## MATERIALS AND METHODS

### Cell culture and reagents

Human gastric cancer cell line SGC-7901 and human normal gastric epithelial cell line GES were kindly provided by our colleague Yongzhan Nie (the Fourth Military Medical University, Xi'an, China), and maintained in our laboratory. These cells were cultured in RPMI-1640 medium (Thermo Scientific, Waltham, MA) supplemented with 10% fetal bovine serum (FBS, HyClone, Thermo Scientific) and 1% penicillin/streptomycin. For hypoxia treatment, cells were grown in a hypoxic chamber (1% O_2_, 5% CO_2_). Lentiviral particles expressing both luc and GFP were purchased from GenTarget (San Diego, CA). Both NIRF heptamethine carbocyanine MHI-148 dye and IR-783-gemcitabine (NIRG) were kindly provided by Leland W.K Chung (Cedars-Sinai Medical Center, Los Angeles, USA) and synthesized as described previously [8, 12]. Bromosulfophthalein (BSP) was purchased from Sigma-Aldrich (St. Louis, MO). D-Luciferin sodium salt was purchased from Gold Biotechnology (Olivette, MO).

### Analysis of NIRF dye uptake in cancer cell models

Gastric cancer cells and normal gastric epithelial cells were incubated with MHI-148 at a concentration of 5 μM at 37°C for 10 min and washed twice with PBS to remove excess dye. Cells were fixed in 10% formaldehyde and subjected to analysis of NIRF dye uptake by a NIR fluorescence microscopy (Olympus 1×71, Olympus, Melville, NY) equipped with a 75 W Xenon lamp and an indo-cyanine green filter cube (excitation/emission: 750-800/820-860 nm) following a protocol as described previously [8, 12]. Cellular NIRF intensity was quantified by ImageJ software (NIH, Bethesda, MD).

### Clinical specimens

All gastric cancer patient specimens were obtained from the Xijing Hospital of Digestive Diseases, and the use of human tissue specimens in research was approved by the institutional review board (IRB) of the Fourth Military Medical University (FMMU).

### Animal studies

Male 6- to 8-week-old BALB/c nude mice were purchased from Vital River (Beijing, China) and raised in the SPF system of the FMMU Laboratory Animal Center. All animal experiments were performed following a protocol (No. 14013) reviewed and approved by the FMMU Institutional Animal Care and Use Committee (IACUC). Mice were anesthetized with ketamine (5 mg/kg) and diazepam (0.25 mg/kg) intraperitoneally, and maintained under isoflurane during surgery and imaging.

To establish subcutaneous and orthotopic tumor xenograft models, different cultured gastric cancer cells were implanted into mice (n=5 for each group) following a published protocol [35].

To establish PDX models of human gastric cancer, clinical tumor samples resected from gastric cancer patients were put into culture media at 4°C immediately and divided into two parts, with one fixed by 4% polyoxymethylene (POM) and the other cut into 1×1×3 mm^3^ small blocks and mixed with matrigel (BD Biosciences, Franklin Lakes, NJ). Mice were anaesthetized and the kidney was exposed via a 1-cm incision on the left or right flank, followed by the transplantation of fresh gastric cancer specimens mixed with matrigel into the subrenal capsule to establish the PDX model (n=5 for each group). Each tumor sample obtained from a patient was cut into 5 pieces and implanted into 5 mice.

To establish gastritis in mice, a gastric ulcer was induced by serosal application of acetic acid according to a published protocol [36].

### Bioluminescence and NIRF imaging for tumor xenograft models

Mice bearing xenograft tumors were injected intraperitoneally with MHI-148 at a dose of 50 nmol/mouse. Whole-body or organ-specific optical imaging was taken at 24 hr using a Caliper Lumina XR Imaging System (PerkinElmer, Waltham, MA) equipped with near-infrared fluorescent filter sets (excitation/emission, 783/840 nm) following a previously described protocol [8, 13]. Part of each piece of tumor tissue was processed as a frozen section and subjected to NIRF microscopy using a 75 W Xenon lamp and an indo-cyanine green filter cube (excitation/emission: 750-800/820-860 nm) as described above. Bioluminescence imaging of luciferase-tagged SGC-7901 tumor xenografts either *in vivo* or *ex vivo* after mice received D-luciferin (3 mg/mouse via intraperitoneal delivery) was performed on a Xenogen IVIS Spectrum Imaging System (PerkinElmer).

### NIRF imaging of clinical samples

Gastric cancer patients underwent surgical resection of tumor tissues, which were immediately perfused with 20 μM MHI-148 solution through residual venous of the dissected tumor tissue, and soaked the tissue for 10 min, then washed with PBS. Tumor tissues were subjected to *ex vivo* NIRF imaging as described above. Part of each tumor tissue was fixed in 4% formaldehyde and embedded in paraffin for subsequent H&E and IHC analyses.

### Pathologic analysis of PDX and clinical tumor specimens

Mice were measured by NIRF imaging every 5 days with a region of interest focused on the renal position. When an obvious bulge formed at the kidney, 3×3×3 mm^3^ tumor tissue was resected under anesthesia and then subcutaneously implanted into another mouse. The tumor obtained from the first subcutaneous passaging was designated as P1. After 3 consecutive subcutaneous passages, the tumor tissue was divided into two parts. One part was fixed with 4% POM to be processed as paraffin-embedded tumor blocks and analyzed for morphology compared to the original tumor sample obtained from the patient by H&E staining. The other part was preserved with liquid nitrogen for further studies. Part of the P3 tumor tissue derived from the PDX model was scissored and digested with 0.25% trypsin at 37°C for 15 min, followed by the addition of RPMI-1640 medium containing 20% FBS for culture at 37°C with 5% CO_2_. After 48 hr, the culture medium was changed to obtain purified primary tumor cells.

### Immunohistochemical analysis

Partial PDX tissue specimens were fixed by polyformaldehyde and embedded in paraffin for subsequent IHC analysis. Tumor sections were stained with antibodies specific for HIF1α (1:100; Abcam, Cambridge, MA), OATP1B3 (1:30; GeneTex, Irvine, CA), CEA (Abcam), or CK8/18 (Abcam) by a previously described protocol [22, 37].

### Quantitative real-time PCR

Total RNA from fresh tumor tissues was isolated using a RNAsimple Total RNA Kit (Tiangen, Shanghai, China) and reverse-transcribed to cDNA using a ReverTra Ace qPCR RT Kit (Toyobo, Osaka, Japan). The transcript levels of select members of the *OTAP* gene family were determined in both cultured tumor cells and tumor tissues. PCR was conducted using SYBR Green PCR Master Mix on an Applied Biosystems StepOnePlus Real-Time PCR System (Thermo Scientific). All primer sequences were used as follows: *OATP1B1* forward 5′-GTCACCATCCTGGAGCTGTT-3′, reverse 5′-GAAGGCCGTGTTGACGATAC-3′; *OATP1B3* forward 5′-GGGTGAATGCCCAAGAGATA-3′, reverse 5′-ATTGACTGGAAACCCATTGC-3′; *OATP2B1* forward 5′-TCAAGCTGTTCGTTCTGTGC-3′, reverse 5′-GTGTTCCCCACCTCGTTGAA-3′; *OATP3A1* forward 5′-TGAGCCAGTCTGTGGATCAG-3′, reverse 5′-ATCACTTGGCGACTTTGGAC-3′; *OATP4A1* forward 5′-CTGCCAGCCAGAACACTACA-3′, reverse 5′- AGAAGGAGGGGCTTTCTCTG-3′; *GAPDH* forward 5′-GACAACAGCCTCAAGATCATCAG-3′, reverse 5′-ATGGCATGGACTGTGGTCATGAG-3′.

### Microarray data sets

Four gastric cancer DNA microarray data sets, Cui [38], DErrico [39], Cho [40] and Wang [41], were downloaded directly from the Oncomine database by licensed access. Microarray data of the Cui, DErrico, Cho and Wang data sets are also publicly available in the Gene Expression Omnibus as GSE27342, GSE13911, GSE13861 and GSE19826, respectively.

### Statistical analysis

All values in figures are presented as the mean ± SD from at least three independent experiments. Data were analyzed by Student's *t*-test (two groups). *P-*values of ≤0.05 (two-sided) were considered to be statistically significant.

## SUPPLEMENTARY MATERIALS FIGURES


