# Supplementary material for: Optical imaging of gastric cancer with near-infrared heptamethine carbocyanine fluorescence dyes
Source: Oncotarget. 2016 Jun 14;7(35):57277–89. doi: 10.18632/oncotarget.10031 (PMC5302988; doi:10.18632/oncotarget.10031)
Supplement: Supplementary file 1 [file oncotarget-07-57277-s001.pdf]

Optical imaging of gastric cancer with near-infrared heptamethine carbocyanine fluorescence dyes

SUPPLEMENTARY FIGURES

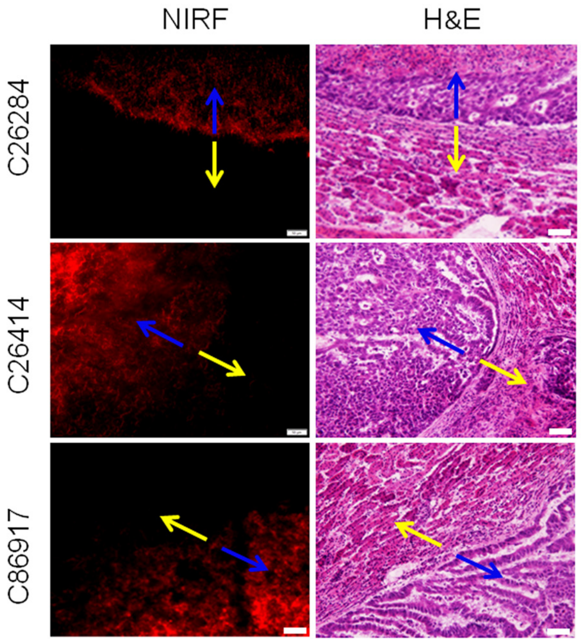

Supplementary Figure S1: H&E and NIRF analyses of PDX tumors. The histology and NIRF dye uptake in three subrenal PDX tumors, C26284, C26414 and C86917, were analyzed in parallel by a NIRF microscopy (left panels) and H&E staining (right panels), respectively. Representative images examining the tumor-kidney interface of PDX samples are shown. Blue and yellow arrows indicate tumor and normal kidney areas, respectively, which indicates that only the xenograft tumor uptake the dye but not the mouse kidney. The Original magnification: x200; scale bars represent 50 μm.

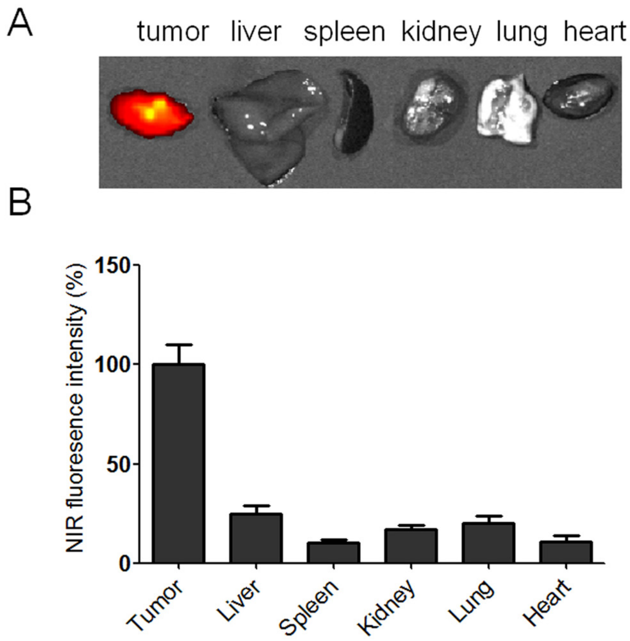

Supplementary Figure S2: Determination of the dye uptake in tumor and select vital organs. A. Mice bearing a subcutaneous PDX-derived gastric tumor was injected with MHI-148 intraperitoneally at a dose of 50 nmol/mouse. 24 hr later, the uptake of NIRF dye in tumor and select organs, including liver, spleen, kidney, lung and heart, dissected from mice were analyzed by *ex vivo* NIRF imaging. Images obtained from a representative mouse are shown. **B.** Quantification of NIRF signal intensity in A. Data are presented as the percentage (mean ± SD, n=5) of signal intensity as normalized to tumor. Signal intensity in tumor was set as 100%.
